# Supplementary figures and images for: Comparative effectiveness of non-pharmacological therapies for postoperative cognitive dysfunction: Protocol for a systematic review and network meta-analysis
Source: PLoS One. 2024 Dec 19;19(12):e0309605. doi: 10.1371/journal.pone.0309605 (PMC11658573; doi:10.1371/journal.pone.0309605)

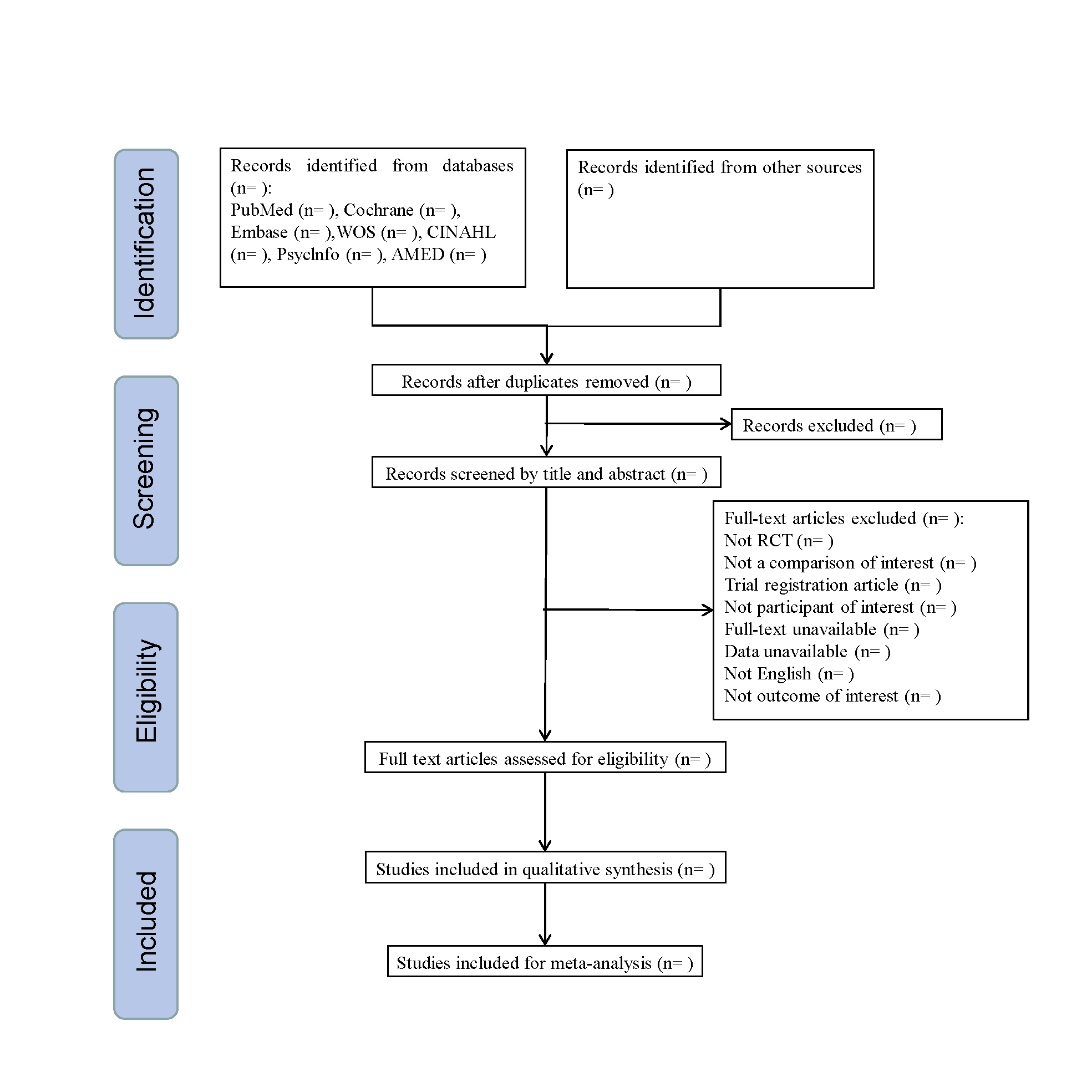

Supplement: S1 Fig — (TIF) [file pone.0309605.s002.tif]
