# Supplementary material for: Comparative effectiveness of non-pharmacological therapies for postoperative cognitive dysfunction: Protocol for a systematic review and network meta-analysis
Source: PLoS One. 2024 Dec 19;19(12):e0309605. doi: 10.1371/journal.pone.0309605 (PMC11658573; doi:10.1371/journal.pone.0309605)
Supplement: S1 File — (DOCX) [file pone.0309605.s003.docx]

**S1 File. Search strategies of each database.**

**PubMed：**

#1 "postoperat*"[Title/Abstract] OR "*surgery"[Title/Abstract] OR "*surgical"[Title/Abstract] OR "*operative"[Title/Abstract] OR "*operation"[Title/Abstract] OR "anesthesia"[Title/Abstract]

#2 "cognitive"[Title/Abstract] OR "cognition"[Title/Abstract] OR "memory"[Title/Abstract] OR "neurocognit*"[Title/Abstract] OR "intelligence"[Title/Abstract] OR "MMSE"[Title/Abstract] OR "MOCA"[Title/Abstract]

#3 #1 AND #2

#4 "postoperative cognitive complications"[MeSH Terms] OR "POCD"[Title/Abstract] OR "postoperative cognitive"[Title/Abstract]

#5 #3 OR #4

#6 "cognitive behavioral therapy"[MeSH Terms] OR "acupuncture therapy"[MeSH Terms] OR "electroacupuncture"[MeSH Terms] OR "exercise therapy"[MeSH Terms] OR "music therapy"[MeSH Terms] OR "psychotherapy"[MeSH Terms] OR "neurofeedback"[MeSH Terms] OR "nonpharmacological"[Title/Abstract] OR "nondrug"[Title/Abstract] OR "cognitive behavioral therapy"[Title/Abstract] OR "cognitive therapy"[Title/Abstract] OR "acupuncture"[Title/Abstract] OR "electroacupuncture"[Title/Abstract] OR "acupoint"[Title/Abstract] OR "high pressure oxygen"[Title/Abstract] OR "biofeedback"[Title/Abstract] OR "rehabilitation"[Title/Abstract] OR "electrical stimulation"[Title/Abstract] OR "nerve stimulation"[Title/Abstract] OR "magnetic stimulation"[Title/Abstract] OR "training"[Title/Abstract] OR "exercise"[Title/Abstract] OR "music"[Title/Abstract] OR "psychotherapy"[Title/Abstract]

#7 "Randomized Controlled Trials as Topic"[MeSH Terms] OR "random allocation"[MeSH Terms] OR "randomized controlled trial"[Publication Type] OR "controlled clinical trial"[Publication Type] OR "clinical trial"[Publication Type] OR "clinical study"[Title/Abstract] OR "trial"[Title/Abstract] OR "placebo"[Title/Abstract] OR "random*"[Title/Abstract]

#8 #5 AND #6 AND #7

**EMBASE**

#1 ‘postoperation’:ti,ab,kw OR ‘postoperative’:ti,ab,kw OR ‘surgery’:ti,ab,kw OR ‘surgical’:ti,ab,kw OR ‘operative’:ti,ab,kw OR ‘operation’:ti,ab,kw OR ‘anesthesia’:ti,ab,kw

#2 ‘cognitive’:ti,ab,kw OR ‘cognition’:ti,ab,kw OR ‘memory’:ti,ab,kw OR ‘neurocognitive’:ti,ab,kw OR ‘neurocognition’:ti,ab,kw OR ‘intelligence’:ti,ab,kw OR ‘MMSE’:ti,ab,kw OR ‘MOCA’:ti,ab,kw

#3 #1 AND #2

#4 ‘postoperative cognitive complications’/exp/mj OR ‘POCD’:ti,ab,kw OR ‘postoperative cognitive’:ti,ab,kw

#5 #3 OR #4

#6 ‘cognitive behavioral therapy’/exp/mj OR ‘acupuncture therapy’/exp/mj OR ‘electroacupuncture’/exp/mj OR ‘exercise therapy’/exp/mj OR ‘music therapy’/exp/mj OR ‘psychotherapy’/exp/mj OR ‘neurofeedback’/exp/mj OR ‘nonpharmacological’:ti,ab,kw OR ‘nondrug’:ti,ab,kw OR ‘cognitive behavioral therapy’:ti,ab,kw OR ‘cognitive therapy’:ti,ab,kw OR ‘acupuncture’:ti,ab,kw OR ‘electroacupuncture’:ti,ab,kw OR ‘acupoint’:ti,ab,kw OR ‘high pressure oxygen’:ti,ab,kw OR ‘biofeedback’:ti,ab,kw OR ‘rehabilitation’:ti,ab,kw OR ‘electrical stimulation’:ti,ab,kw OR ‘nerve stimulation’:ti,ab,kw OR ‘magnetic stimulation’:ti,ab,kw OR ‘training’:ti,ab,kw OR ‘exercise’:ti,ab,kw OR ‘music’:ti,ab,kw OR ‘psychotherapy’:ti,ab,kw

#7 ‘Randomized Controlled Trials as Topic’/exp/mj OR ‘random allocation’/exp/mj OR ‘clinical study’:ti,ab,kw OR ‘trial’:ti,ab,kw OR ‘placebo’:ti,ab,kw OR ‘randomly’:ti,ab,kw OR ‘randomized’:ti,ab,kw

#8 #5 AND #6 AND #7

**Web of scienc**

TS=(((‘postoperat*’ OR ‘*surgery’ OR ‘*surgical’ OR ‘*operative’ OR ‘*operation’ OR ‘anesthesia’) AND (‘cognitive’ OR ‘cognition’ OR ‘memory’ OR ‘neurocognit*’ OR ‘intelligence’ OR ‘MMSE’ OR ‘MOCA’) OR (‘postoperative cognitive complications’ OR ‘POCD’ OR ‘postoperative cognitive’))) AND TS=(‘nonpharmacological’ OR ‘nondrug’ OR ‘cognitive behavioral therapy’ OR ‘cognitive therapy’ OR ‘acupuncture’ OR ‘electroacupuncture’ OR ‘acupoint’ OR ‘psychotherapy’ OR ‘neurofeedback’ OR ‘high pressure oxygen’ OR ‘biofeedback’ OR ‘rehabilitation’ OR ‘electrical stimulation’ OR ‘nerve stimulation’ OR ‘magnetic stimulation’ OR ‘training’ OR ‘exercise’ OR ‘music’) AND TS=(‘Randomized Controlled Trials as Topic’ OR ‘random allocation’ OR ‘clinical study’ OR ‘trial’ OR ‘placebo’ OR ‘random*’)

**Cochrane Library**

#1 postoperat*:ti,ab,kw OR *surgery:ti,ab,kw OR *surgical:ti,ab,kw OR *operative:ti,ab,kw OR *operation:ti,ab,kw OR anesthesia:ti,ab,kw

#2 cognitive:ti,ab,kw OR cognition:ti,ab,kw OR memory:ti,ab,kw OR neurocognit*:ti,ab,kw OR intelligence:ti,ab,kw OR MMSE:ti,ab,kw OR MOCA:ti,ab,kw

#3 #1 AND #2

#4 POCD:ti,ab,kw OR postoperative cognitive:ti,ab,kw

#5 Mesh descriptor: [postoperative cognitive complications] explode all trees

#6 #3 OR #4 OR #5

#7 Mesh descriptor: [cognitive behavioral therapy] explode all trees

#8 Mesh descriptor: [acupuncture therapy] explode all trees

#9 Mesh descriptor: [electroacupuncture] explode all trees

#10 Mesh descriptor: [exercise therapy] explode all trees

#11 Mesh descriptor: [music therapy] explode all trees

#12 Mesh descriptor: [psychotherapy] explode all trees

#13 Mesh descriptor: [neurofeedback] explode all trees

#14 nonpharmacological:ti,ab,kw OR nondrug:ti,ab,kw OR "cognitive behavioral therapy":ti,ab,kw OR "cognitive therapy":ti,ab,kw OR acupuncture:ti,ab,kw OR electroacupuncture:ti,ab,kw OR acupoint:ti,ab,kw OR "high pressure oxygen":ti,ab,kw OR biofeedback:ti,ab,kw OR rehabilitation:ti,ab,kw OR "electrical stimulation":ti,ab,kw OR "nerve stimulation":ti,ab,kw OR "magnetic stimulation":ti,ab,kw OR training:ti,ab,kw OR exercise:ti,ab,kw OR music:ti,ab,kw OR psychotherapy:ti,ab,kw

#15 #7 OR #8 OR #9 OR #10 OR #11 OR #12 OR #13 OR #14

#16 Mesh descriptor: [Randomized Controlled Trials as Topic] explode all trees

#17 Mesh descriptor: [random allocation] explode all trees

#18 clinical study:ti,ab,kw OR trial:ti,ab,kw OR placebo:ti,ab,kw OR random*:ti,ab,kw

#19 #16 OR #17 OR #18

#20 #6 AND #15 AND #19

**CINAHL, AMED, and PsycINFO**

S1 SU ( postoperat* OR *surgery OR *surgical OR *operative OR *operation OR anesthesia ) AND SU ( cognitive OR cognition OR memory OR neurocognit* OR intelligence OR MMSE OR MOCA )

S2 SU POCD OR SU 'postoperative cognitive'

S3 S1 OR S2

S4 ((((DE "ACUPUNCTURE") OR (DE "ELECTROACUPUNCTURE")) OR (DE "EXERCISE therapy")) OR (DE "MUSIC therapy")) OR (DE "PSYCHOTHERAPY")

S5 SU nonpharmacological OR nondrug OR 'cognitive behavioral therapy' OR 'cognitive therapy' OR acupuncture OR electroacupuncture OR acupoint OR 'high pressure oxygen' OR biofeedback OR rehabilitation OR 'electrical stimulation' OR 'nerve stimulation' OR 'magnetic stimulation' OR training OR exercise OR music OR psychotherapy

S6 S4 OR S5

S7 DE "RANDOMIZED controlled trials"

S8 SU clinical study OR trial OR placebo OR random*

S9 S7 OR S8

S10 S3 AND S6 AND S9
