# Supplementary material for: Comparative effectiveness of non-pharmacological therapies for postoperative cognitive dysfunction: Protocol for a systematic review and network meta-analysis
Source: PLoS One. 2024 Dec 19;19(12):e0309605. doi: 10.1371/journal.pone.0309605 (PMC11658573; doi:10.1371/journal.pone.0309605)
Supplement: Attachment — Submitted filename: Response to Reviewers and Editors.docx [file pone.0309605.s004.docx]

Dear Editors and Reviewers:

We sincerely appreciate the editor's and reviewers’ comments concerning our manuscript entitled “Comparative effectiveness of non-pharmacological therapies for postoperative cognitive dysfunction: Protocol for a systematic review and network meta-analysis”. Those comments are all valuable and very helpful for revising and improving our paper, as well as the important guiding significance to our review. We have studied the comments carefully and have made corrections that we hope meet PLOS ONE’s publication criteria. These changes are indicated using text in the red font in the revised manuscript. The main corrections in the paper and the responses to the editor and reviewers’ comments are as follows:

**Editor’s Comments:**

David Chau Academic Editor:

1. Please ensure that your manuscript meets PLOS ONE's style requirements, including those for file naming. The PLOS ONE style templates can be found at

**Response:** Thank you very much for your thoughtful feedback. We have thoroughly reviewed the PLOS ONE style templates you graciously provided and have meticulously revised our manuscript to adhere to the specified requirements. This includes adjustments to file naming, title, authorship details, affiliations, and the main body of the text. We greatly appreciate your guidance in ensuring compliance with the journal's guidelines.

2. We note that the grant information you provided in the ‘Funding Information’ and ‘Financial Disclosure’ sections do not match.

When you resubmit, please ensure that you provide the correct grant numbers for the awards you received for your study in the ‘Funding Information’ section.

**Response:** Thank you for your valuable input. We have carefully reviewed and updated the grant information in the 'Funding Information' section as per your suggestion. This study received financial support from the State Administration of Traditional Chinese Medicine, under the National Key Research and Development Program of China (Grant No. 2019YFC1709700), the National Natural Science Foundation of China (Grants Nos. 81973961, 82004486), and the Project of the Science and Technology Department of Sichuan Province (Grant No. 2021YFS0087). Fanrong Liang acknowledges support from these organizations as a grantee.

3. Thank you for stating the following in the Acknowledgments Section of your manuscript:

"The authors thank the State Administration of Traditional ChineseMedicine, the National Natural Science Foundation of China and theScience and Technology Department of Sichuan Province for the financial support of the project. We also thank editage for editing the English text of the draft article."

We note that you have provided funding information that is not currently declared in your Funding Statement. However, funding information should not appear in the Acknowledgments section or other areas of your manuscript. We will only publish funding information present in the Funding Statement section of the online submission form.

Please remove any funding-related text from the manuscript and let us know how you would like to update your Funding Statement. Currently, your Funding Statement reads as follows:

"This review will be financially supported by the State Administration of Traditional Chinese Medicine, National key research and development program of China (No. 2019YFC1709700), the National Natural Science Foundation of China (Nos. 81973961, 82004486), and the Project of Science and Technology Department of Sichuan Province (No. 2021YFS0087)."

Please include your amended statements within your cover letter; we will change the online submission form on your behalf.

**Response:** Thanks for your significant comment. We have removed the funding information from the manuscript and added the amended statement in the cover letter.

4. Thank you for stating the following financial disclosure:

"This review will be financially supported by the State Administration of Traditional Chinese Medicine, National key research and development program of China (No. 2019YFC1709700), the National Natural Science Foundation of China (Nos. 81973961, 82004486), and the Project of Science and Technology Department of Sichuan Province (No. 2021YFS0087)."

Please state what role the funders took in the study. If the funders had no role, please state: ""The funders had no role in study design, data collection and analysis, decision to publish, or preparation of the manuscript.""

If this statement is not correct you must amend it as needed.

Please include this amended Role of Funder statement in your cover letter; we will change the online submission form on your behalf.

**Response:** Thanks for your significant comment. The funders had no role in study design, data collection and analysis, decision to publish, or preparation of the manuscript. We have added the amended Role of Funder statement in the cover letter.

5. When completing the data availability statement of the submission form, you indicated that you will make your data available on acceptance. We strongly recommend all authors decide on a data sharing plan before acceptance, as the process can be lengthy and hold up publication timelines. Please note that, though access restrictions are acceptable now, your entire data will need to be made freely accessible if your manuscript is accepted for publication. This policy applies to all data except where public deposition would breach compliance with the protocol approved by your research ethics board. If you are unable to adhere to our open data policy, please kindly revise your statement to explain your reasoning and we will seek the editor's input on an exemption. Please be assured that, once you have provided your new statement, the assessment of your exemption will not hold up the peer review process.

**Response:** Thanks for your significant recommendation. All authors have decided on a data sharing plan before acceptance.

6. We notice that your supplementary figures are uploaded with the file type 'Figure'. Please amend the file type to 'Supporting Information'. Please ensure that each Supporting Information file has a legend listed in the manuscript after the references list.

[Note: HTML markup is below. Please do not edit.]

**Response:** Thanks for your suggestion, we have amended the file type to "Supporting Information", and we have listed a legend in the manuscript.

**Reviewers' comments:**

Reviewer's Responses to Questions

Comments to the Author

1. Does the manuscript provide a valid rationale for the proposed study, with clearly identified and justified research questions?
The research question outlined is expected to address a valid academic problem or topic and contribute to the base of knowledge in the field.

Reviewer #1: No

Reviewer #2: Yes

Reviewer #3: Yes

2. Is the protocol technically sound and planned in a manner that will lead to a meaningful outcome and allow testing the stated hypotheses?
The manuscript should describe the methods in sufficient detail to prevent undisclosed flexibility in the experimental procedure or analysis pipeline, including sufficient outcome-neutral conditions (e.g. necessary controls, absence of floor or ceiling effects) to test the proposed hypotheses and a statistical power analysis where applicable. As there may be aspects of the methodology and analysis which can only be refined once the work is undertaken, authors should outline potential assumptions and explicitly describe what aspects of the proposed analyses, if any, are exploratory.

Reviewer #1: No

Reviewer #2: Yes

Reviewer #3: Yes

3. Is the methodology feasible and described in sufficient detail to allow the work to be replicable?
Descriptions of methods and materials in the protocol should be reported in sufficient detail for another researcher to reproduce all experiments and analyses. The protocol should describe the appropriate controls, sample size calculations, and replication needed to ensure that the data are robust and reproducible.

Reviewer #1: No

Reviewer #2: Yes

Reviewer #3: Yes

4. Have the authors described where all data underlying the findings will be made available when the study is complete?

The [PLOS Data policy](https://journals.plos.org/plosone/s/materials-and-software-sharing) requires authors to make all data underlying the findings described in their manuscript fully available without restriction, with rare exception, at the time of publication. The data should be provided as part of the manuscript or its supporting information, or deposited to a public repository. For example, in addition to summary statistics, the data points behind means, medians and variance measures should be available. If there are restrictions on publicly sharing data—e.g. participant privacy or use of data from a third party—those must be specified.

Reviewer #1: Yes

Reviewer #2: Yes

Reviewer #3: Yes

5. Is the manuscript presented in an intelligible fashion and written in standard English?
PLOS ONE does not copyedit accepted manuscripts, so the language in submitted articles must be clear, correct, and unambiguous. Any typographical or grammatical errors should be corrected at revision, so please note any specific errors here.

Reviewer #1: No

Reviewer #2: Yes

Reviewer #3: Yes

6. Review Comments to the Author

Please use the space provided to explain your answers to the questions above and, if applicable, provide comments about issues authors must address before this protocol can be accepted for publication. You may also include additional comments for the author, including concerns about research or publication ethics.

You may also provide optional suggestions and comments to authors that they might find helpful in planning their study.


(Please upload your review as an attachment if it exceeds 20,000 characters)

**Reviewer #1:**

1.You need to define POCD correctly. Do you mean POCD or delirium? Some of the definitions, I think you are talking about delirium and in some of them you are talking about POCD (Delirium is short term (up to 7 days after surgery, POCD occurs later than this). Please decide whether the drive is POCD or delirium and modify / re-write all of your thinking, background and methods accordingly. The mechanistic impact of interventions on these two processes is likely to be different (as are the causes).

**Response:** Thank you for your valuable comments. The participants of this study are postoperative cognitive dysfunction (POCD) patients, not postoperative delirium patients. According to the 2018 Naming Consensus on Surgery-Related and Anesthesia-Related Cognitive Injury, ‘postoperative delirium’ is defined as that which occurs in hospital up to 1 week postprocedure or until discharge (whichever occurs first) and meets DSM-5 diagnostic criteria. On the other hand, POCD refers to cognitive impairment present in patients from 30 days to 1 year after surgery.

However, recognizing the disparities between the guidelines' definition and the actual definitions of POCD in RCTs, particularly considering literature predating 2018, which did not explicitly define the timeframe for POCD, we've deliberated on this issue. Consequently, this study will include patients with cognitive decline up to 1 year postoperatively, diagnosed explicitly as POCD in RCTs. Some modifications have been implemented to the introduction and inclusion criteria sections accordingly. Please refer to the manuscript for details.

Page 5, lines 63-67;

*Postoperative cognitive dysfunction (POCD) is a common postoperative complication of the central nervous system and is defined as cognitive decline occurring within 1 year after a surgery (1, 2). This decline is characterized by a decrease in memory, abstraction, and attention (3).*

Page 9, lines 135-143;

*This study will include patients who underwent surgery under general or epidural anaesthesia, regardless of the surgery type, with no limitations on the participant's sex, race, or degree of education. We will exclude patients aged < 18 years and those with unstable vital signs. To reduce clinical heterogeneity, we will also exclude patients with diagnoses of postoperative delirium, pre-existing cognitive impairment or mental disorders before the surgery, and cognitive dysfunction occurring more than 1 year after the surgery (27).*

2. Why would you include cross-over trials?

**Response:** Thank you for your feedback. Initially, our plan included crossover trials with Phase I outcomes available, as the initial part of the study is influenced by a single intervention/control method, and results after crossover intervention would not be included. However, upon further consideration and taking into account your suggestion, and also considering that no existing crossover trials on POCD were found in our actual search process, so we have excluded crossover trials from our study. Please refer to the manuscript for details.

Page 8, lines 129-134;

*All randomised controlled trials of non-pharmacological interventions for postoperative cognitive function will be included. Non-randomised controlled trials, crossover trials, cohort studies, reviews, case reports, expert experience, conference papers, animal studies, and duplicate publications will be excluded.*

3. Types of participants - I would consider stratifying/excluding by surgery severity as there is likely to be no meaningful answer as the impact of different predisposing factors is different. i.e. the things that cause / impact delirium for people having GA hernia repair is different to cardiac surgery etc.... (also what does "undergoing surgery for more than 1 year" mean - I assume this is a typo?)

**Response:** Thank you for your suggestion. As you pointed out, there may be no meaningful answer for different surgical severity because of the different effects of different preconditions. Therefore, in this study, we will conduct a reasonable subgroup analysis according to the intervention timing, type of surgery, average age of the participant, and so on.

We have decided not to include a category for "more than 1 year after surgery" as POCD is defined as cognitive impairment present during the 1-year post-surgery period. Cognitive impairment occurring more than 1 year post-surgery may be considered mild cognitive impairment rather than POCD. Please refer to the manuscript for details.

Page 17, lines 253-261;

*Based on the guidelines from Cochrane Handbook 6.1 (35), when I^2^ ≥50% and P <0.05 (i.e., in case of significant heterogeneity), a subgroup analysis will be performed reasonably according to the intervention timing, type of surgery, and average participant age, among others (if sufficient data are available). Simultaneously, based on the sample size and methodological quality, a sensitivity analysis will be performed by eliminating studies with small sample sizes or a high risk of bias to verify the accuracy and stability of the results.*

Page 9, lines 135-143;

*This study will include patients who underwent surgery under general or epidural anaesthesia, regardless of the surgery type, with no limitations on the participant's sex, race, or degree of education. We will exclude patients aged < 18 years and those with unstable vital signs. To reduce clinical heterogeneity, we will also exclude patients with diagnoses of postoperative delirium, pre-existing cognitive impairment or mental disorders before the surgery, and cognitive dysfunction occurring more than 1 year after the surgery (27).*

4. The types of comparator / control need to be more explicit - what is "waiting list" comparator?

**Response:** Thank you for your suggestion. Considering the current RCT studies, due to the nature of postoperative conditions, all groups receive standard care and treatment, with no specific distinction made between standard care and the non-intervention group. After discussion, we have decided to define the comparison groups/control groups as follows: a. Non-intervention group; b. Routine care group; c. Sham group. We have made some modifications to the inclusion criteria for the control group. Please refer to the manuscript for details.

Pages 9-10, lines 150-153;

*The control group will include the no-intervention, routine care, and sham groups. Patients who were undergoing pharmacological interventions associated with improved cognition will be excluded.*

5. The primary outcome needs to be POCD o delirium (whatever they pick as the driver for this) and not the MMSE as MMSE may not be recorded by some studies - the phenomena of POCD / delirium will be and therefore they will end up having an incomplete review. (MMSE is also a screening test for cognitive impairment and not delirium).

**Response:** Thank you for your valuable suggestion. The participants of this study are patients with postoperative cognitive impairment, not patients with delirium. The selection of MMSE as the primary outcome measure is primarily because it is the most frequently utilized indicator in randomized controlled trials for observing cognitive function changes. Moreover, MMSE is suitable for environments with varying literacy levels. In comparison to POCD incidence, MMSE enables better observation of longitudinal cognitive level changes. Please refer to the manuscript for details.

Page 10, lines 154-163;

*The primary outcome will be changes in the overall cognitive function from the baseline to the end of the intervention; these will be assessed using Mini-Mental State Examination (MMSE) scores. The MMSE, a brief pencil-and-paper cognitive test, is the most widely used measure of cognitive function in clinical, research, and community settings (28, 29). It is well validated in low-literacy settings (30), and is the primary measure of POCD in existing clinical studies. The secondary outcome will be the incidence of POCD, measured according to the criteria of each study.*

6. Search strategy should be in appendix - but is very limited and is likely to miss a number of manuscripts.

**Response:** The full search strategy has been supplemented and uploaded in the S1 File. Search strategies of each database. Please refer to the manuscript for details.

7. It should like they have already started "The output of all searches was imported into the Endnote V.X9 software for management." and "A comparative adjustment funnel plot was used to explore the reported bias and small-scale effects" The protocol should be in the future tense.....and ideally they shouldn't have started

**Response:** Thanks for your suggestion, the modification has been completed. Please refer to the manuscript for details.

8. There is a good deal of the passive tense in the methods - which makes it hard to read.

**Response:** Thanks for your suggestion, the language of the article has been revised. We would like to thank Editage (www.editage.cn) for English language editing.

9. The discussion is extremely short - I know it is a protocol, but I would like some appraisal of the methodology, what definitions they have used, why they chose specific outcomes etc....

**Response:** Thank you for your input. In the discussion section, we provide further elaboration on the selection of methods and results. Please consult the manuscript for detailed information.

Pages 18-19, lines 279-299;

*Many studies have demonstrated the effectiveness of different non-pharmacological therapies for the prevention and treatment of POCD. However, previous meta-analyses have often been limited to a single non-pharmacological therapy, thereby lacking a comprehensive and holistic basis. NMAs emphasize the comparison of multiple interventions under the same conditions, thereby enabling a more thorough and accurate estimation of the treatment effects. This will be the first NMA to compare the effectiveness of four non-pharmacological therapies for postoperative cognitive impairment; its results can help synthesize direct and indirect comparative evidence and will ultimately enable a ranking of these therapies to help clinicians and patients develop intervention plans. In addition, GRADE will be used to evaluate the quality of evidence.*

*This study protocol has some limitations. First, inclusion of different types of non-pharmacological therapies may carry a large risk of heterogeneity, which may impact our results. Second, from the perspective of the study participants, POCD does not have a unified definition; from the perspective of the study results, MMSE scores are not sufficiently sensitive indicators for diagnosing cognitive impairment, and the incidence of POCD is measured according to each study's criteria. In addition, only English-language trials will be selected, which could result in publication bias..*

**Reviewer #2:**

This study protocol, “Comparative effectiveness of non-pharmacological therapies for postoperative cognitive dysfunction: Protocol for a systematic review and network meta-analysis”, describes meta-analysis methods to evaluate and compare four non-pharmacological interventions to prevent/reduce post-surgical cognitive dysfunction. The value of the study lies in its effort to compare non-pharmacological interventions for a very important and relevant topic. It is a well-written proposal with a robust methodology; however, I have a few comments as mentioned below —

1.Pagination and Line Numbers: Include page numbers and line numbers for precise feedback.

**Response:** Many thanks for your review. We have included the page number and line number of the article.

2.Briefly explain non-pharmacological therapies and how they are similar/different from each other. Additionally, provide insight into their current utilization in the field.

**Response:** Many thanks for your review. We have explained the definitions of different non-pharmacological therapies and the differences between them, providing an overview of their use in cognitive disorders. Please refer to the manuscript for details.

Pages 6-7, lines 94-110;

*Acupuncture has been used to treat a variety of diseases, and EA combines the effects of electrical stimulation with those of conventional acupuncture (19). A systematic review (SR) published in 2023 (20) revealed that acupuncture-related techniques, including MA and EA, are associated with fewer postoperative cognitive complications. TEAS, a combination of transcutaneous electrical nerve stimulation and acupoint therapy, has been found to attenuate brain damage by inhibiting oxidative stress responses (21). An SR published in 2022 (22) indicated that TEAS can prevent early postoperative cognitive decline after general anaesthesia in older adults. Cognitive training is the scientific assessment and systematic training of people's cognitive ability. An SR and meta-analysis by Bowden et al. (23) found that cognitive intervention is effective in improving the cognitive function after general surgery. Guidelines for other cognitive disorders (24) have also recommended cognitive stimulation to improve the cognitive function.*

3.Demonstrate, how authors plan to control for effects of medications like anticholinergics that have deleterious effects on cognition.

**Response:** Thank you for your suggestion. The purpose of this study is to investigate the effects of various non-pharmacological interventions on postoperative cognitive dysfunction. If a drug affecting cognitive function is inevitably used, the study will be excluded.

Page 9, lines 147-149;

*If pharmaceutical treatments associated with improved cognition were used, this intervention would be excluded.*

4.Additionally, how would you control for the effect of other non-pharmacological treatments like transcranial magnetic brain stimulation that has been shown to improve cognition?

**Response:** Thank you for your suggestion. The initial search for this study encompassed various non-pharmacological interventions, including transcranial magnetic stimulation, music therapy, and biofeedback therapy, among others. However, to ensure the study's focus and meaningful outcomes, we opted to refine the intervention categories. This decision was informed by current guidelines on non-pharmacological treatments for cognitive disorders and previous literature searches, leading us to narrow down the intervention methods to the four most commonly used ones.

Rodakowski J, Saghafi E, Butters MA, Skidmore ER. Non-pharmacological interventions for adults with mild cognitive impairment and early stage dementia: An updated scoping review. Mol Aspects Med. 2015;43-44:38-53. [doi:10.1016/j.mam.2015.06.003](http://doi.org/10.1016/j.mam.2015.06.003)

Knopman DS, Petersen RC. Mild cognitive impairment and mild dementia: a clinical perspective. Mayo Clin Proc. 2014;89(10):1452-1459. doi:10.1016/j.mayocp.2014.06.019

5. MMSE is a cognitive screening tool or can be used as a diagnostic adjunct but MMSE is not solely and independently used to make clinical diagnosis of cognitive impairment. Besides, MMSE does not identify early-stage cognitive impairment. Explain how authors plan to address this issue sin their outcomes.

**Response:** Thank you for your suggestion. In this study, MMSE will be chosen as the primary outcome measure primarily because it is the most commonly used indicator in RCTs for observing changes in cognitive function. Secondly, MMSE is adaptable in environments with low literacy levels. Furthermore, MMSE is adaptable to environments with low literacy levels and can better track longitudinal changes in cognitive levels compared to the occurrence rate of POCD. However, it is acknowledged in the limitations of this article that MMSE may not be sufficiently sensitive for diagnosing cognitive impairment. Please refer to the manuscript for details.

Page 10, lines 154-163;

*The primary outcome will be changes in the overall cognitive function from the baseline to the end of the intervention; these will be assessed using Mini-Mental State Examination (MMSE) scores. The MMSE, a brief pencil-and-paper cognitive test, is the most widely used measure of cognitive function in clinical, research, and community settings (28, 29). It is well validated in low-literacy settings (30), and is the primary measure of POCD in existing clinical studies. The secondary outcome will be the incidence of POCD, measured according to the criteria of each study.*

6.Briefly explain, especially for new readers, what graphics authors plan to present using the GetData graph digitizer.

**Response:** Thank you for your suggestion. The manuscript provides a brief introduction to the GetData software. Please refer to the manuscript for details.

Pages 14-15, lines 213-216;

*This software can extract and transform data from published images in a rapid manner. In case of missing data, we will contact the corresponding author via email. If data cannot be obtained, we will only analyse the available data.*

7.Briefly explain I^2^ statistics.

**Response:** Thank you for your suggestion, please refer to the Pairwise meta-analysis section in the manuscript for details.

Page 15, lines 220-228;

*We will examine the differences in the participant characteristics, baseline data, intervention measures, and outcomes to assess the presence and degree of clinical heterogeneity across the included studies using I^2^ statistics and p-values. I^2^ values of ＞25%, 50%, and 75% will be considered indicative of low, moderate, and high statistical heterogeneity, respectively. I^2^ values of >50% will be considered indicative of substantial heterogeneity (32), and a random effects model will be used for the meta-analysis; otherwise, a fixed effects model will be used to combine the effect sizes..*

8.Briefly explain heterogeneity in terms of variability in treatment effects across eligible RCTs.

**Response:** Thank you for your suggestion. From the results of the study, it can be seen that there is no unified quantified diagnostic criteria for POCD, and the incidence of POCD is measured based on the standards of individual studies, which may lead to outcome variability. Please refer to the manuscript for details.

Page 10, lines 161-163;

*The secondary outcome will be the incidence of POCD, measured according to the criteria of each study.*

9.Briefly explain the network plot, nodes, lines, and node-splitting model with examples.

**Response:** Thank you for your suggestion. A brief description of the network plot, nodes, lines, and node-splitting model has been provided in the Network meta-analysis section of the manuscript.

Pages 15-16, lines 232-236;

*The larger the nodes, the larger the sample size, and the thicker the lines, the larger the number of RCTS. Each node represents a different intervention, and the connecting lines between nodes represent the effect of comparing the two interventions.*

10.Describe in detail the bias associated with meta-analysis, and how multiple treatment comparison (network) meta-analysis reduces those biases. Explain funnel plot and publication bias.

**Response:** Thank you very much for your suggestion. Network meta-analysis is a method based on multiple studies, used to analyze direct and indirect comparison results between two or more interventions. Network meta-analysis emphasizes comparing multiple interventions under the same conditions to provide a more comprehensive and accurate estimate of treatment effects.

A funnel plot is a simple scatter plot that reflects the estimated intervention effects of individual studies under a certain sample size or precision. It can be used to identify publication bias or other biases, and the advantage of a funnel plot is its intuitiveness. Please refer to the manuscript for details.

Page 18, lines 281-285;

*However, previous meta-analyses have often been limited to a single non-pharmacological therapy, thereby lacking a comprehensive and holistic basis. NMAs emphasize the comparison of multiple interventions under the same conditions, thereby enabling a more thorough and accurate estimation of the treatment effects.*

Page 17, lines 264-268;

*A funnel plot is a simple scatter plot that can be used intuitively to identify publication and other biases. If more than ten studies are included, funnel plots will be used to detect any reported bias. An asymmetrical funnel plot will be considered indicative of publication bias.*

**Reviewer #3:**

The authors have done an excellent job in crafting a manuscript that is both informative and well-written. The study is described in detail and the language used throughout the article is of the highest quality. The topic being addressed is of great relevance to the scientific community and promises to provide valuable insights upon completion. It's clear that the authors have put a lot of hard work and dedication into this study, and they should be applauded for their efforts. Congratulations on a job well done!

**Response:** Thank you very much for this precious approval.
