# Supplementary material for: Comparative effectiveness of non-pharmacological therapies for postoperative cognitive dysfunction: Protocol for a systematic review and network meta-analysis
Source: PLoS One. 2024 Dec 19;19(12):e0309605. doi: 10.1371/journal.pone.0309605 (PMC11658573; doi:10.1371/journal.pone.0309605)
Supplement: Attachment — Submitted filename: Response to Reviewers and Editors.docx [file pone.0309605.s005.docx]

Dear Editors and Reviewers:

We sincerely appreciate the editor's and reviewers’ comments concerning our manuscript entitled “Comparative effectiveness of non-pharmacological therapies for postoperative cognitive dysfunction: Protocol for a systematic review and network meta-analysis”. Those comments are all valuable and very helpful for revising and improving our paper, as well as the important guiding significance to our review. We have studied the comments carefully and have made corrections that we hope meet PLOS ONE’s publication criteria. These changes are indicated using text in the red font in the revised manuscript. The main corrections in the paper and the responses to the editor and reviewers’ comments are as follows:

**Editor’s Comments:**

David Chau Academic Editor:

1. Therefore, we invite you to submit a revised version of the manuscript that addresses the points raised during the review process.

specifically, reviewer-1 considers a start of search and review of your protocol that goes against the prerequests of protocol submission:

<https://journals.plos.org/plosone/s/reviewer-guidelines#loc-study-protocols>

**Response:** Thank you very much for your thoughtful feedback. We have meticulously revised our manuscript to adhere to the specified requirements. n response to reviewer 1's suggestion, we need to make the necessary explanations： The study has not officially begun, but a pre-search of the topics has been conducted to develop a research protocol. We sincerely apologize for any misunderstandings arising from the misuse of tenses in the initial draft. We are committed to embracing the reviewer's recommendations with an open mind and we greatly appreciate your guidance in ensuring compliance with the journal's guidelines.

**Reviewers' comments:**

Reviewer's Responses to Questions

Comments to the Author

1. Does the manuscript provide a valid rationale for the proposed study, with clearly identified and justified research questions?

The research question outlined is expected to address a valid academic problem or topic and contribute to the base of knowledge in the field.

Reviewer #1: No

Reviewer #2: Yes

Reviewer #3: Yes

2. Is the protocol technically sound and planned in a manner that will lead to a meaningful outcome and allow testing the stated hypotheses?

The manuscript should describe the methods in sufficient detail to prevent undisclosed flexibility in the experimental procedure or analysis pipeline, including sufficient outcome-neutral conditions (e.g. necessary controls, absence of floor or ceiling effects) to test the proposed hypotheses and a statistical power analysis where applicable. As there may be aspects of the methodology and analysis which can only be refined once the work is undertaken, authors should outline potential assumptions and explicitly describe what aspects of the proposed analyses, if any, are exploratory.

Reviewer #1: No

Reviewer #2: Yes

Reviewer #3: Yes

3. Is the methodology feasible and described in sufficient detail to allow the work to be replicable?

Descriptions of methods and materials in the protocol should be reported in sufficient detail for another researcher to reproduce all experiments and analyses. The protocol should describe the appropriate controls, sample size calculations, and replication needed to ensure that the data are robust and reproducible.

Reviewer #1: No

Reviewer #2: Yes

Reviewer #3: Yes

4. Have the authors described where all data underlying the findings will be made available when the study is complete?

The [PLOS Data policy](https://journals.plos.org/plosone/s/materials-and-software-sharing) requires authors to make all data underlying the findings described in their manuscript fully available without restriction, with rare exception, at the time of publication. The data should be provided as part of the manuscript or its supporting information, or deposited to a public repository. For example, in addition to summary statistics, the data points behind means, medians and variance measures should be available. If there are restrictions on publicly sharing data—e.g. participant privacy or use of data from a third party—those must be specified.

Reviewer #1: Yes

Reviewer #2: Yes

Reviewer #3: Yes

5. Is the manuscript presented in an intelligible fashion and written in standard English?

PLOS ONE does not copyedit accepted manuscripts, so the language in submitted articles must be clear, correct, and unambiguous. Any typographical or grammatical errors should be corrected at revision, so please note any specific errors here.

Reviewer #1: No

Reviewer #2: Yes

Reviewer #3: Yes

6. Review Comments to the Author Please use the space provided to explain your answers to the questions above and, if applicable, provide comments about issues authors must address before this protocol can be accepted for publication. You may also include additional comments for the author, including concerns about research or publication ethics.

You may also provide optional suggestions and comments to authors that they might find helpful in planning their study.

Reviewer #1: The authors have already started the search and review process - therefore there is little point in reviewing a protocol to which the authors have already started (see point 7 in 1st review) and their response to point 2 in this review in which they say they have actually started searching and so they have excluded cross - over studies.

You can't exclude patients with a diagnosis of delirium in your exclusion criteria as delirium is closely associated with cognitive dysfunction. You have to choose your outcome measure that means you are measuring POCD and not delirium

You cannot review a protocol where the work has already started as there is little point in providing any feedback.

**Response:** Thank you for your feedback. This study has conducted a preliminary search to determine the retrieval formula. The research has not yet started, so we will also sincerely learn and accept your opinions. As you said, postoperative delirium (POD) is closely related to postoperative cognitive impairment. POD is defined as the clinical syndrome of transient fluctuating disturbance in attention, mental status, and consciousness, occurring immediately postoperatively after anesthesia and surgery up to during hospitalization(1, 2). Unlike POD being diagnosed with clinical symptoms, POCD is defined by neuropsychological tests including different domains of impaired cognition, such as verbal memory, visual memory, language comprehension, visuospatial abstraction, attention, and concentration(3). Before 2018, the standard for POCD was limited to research. There was no standardized definition.(4). In this study, acknowledging the availability of well-defined neuropsychological scales for the delineation and diagnosis of POCD, as well as the established diagnostic criteria for POCD within the framework of guideline definitions and RCTs, the Mini-Mental State Examination (MMSE) was selected as the primary outcome measure. Concurrently, the incidence rate of POCD was designated as the secondary outcome measure.

1.	Newman MF, Kirchner JL, Phillips-Bute B, Gaver V, Grocott H, Jones RH, et al. Longitudinal assessment of neurocognitive function after coronary-artery bypass surgery. The New England Journal of Medicine. 2001;344(6):395-402.

2.	Robinson TN, Raeburn CD, Tran ZV, Angles EM, Brenner LA, Moss M. Postoperative delirium in the elderly: risk factors and outcomes. Ann Surg. 2009;249(1):173-8. http://doi.org/10.1097/SLA.0b013e31818e4776

3.	Moller JT, Cluitmans P, Rasmussen LS, Houx P, Rasmussen H, Canet J, et al. Long-term postoperative cognitive dysfunction in the elderly ISPOCD1 study. ISPOCD investigators. International Study of Post-Operative Cognitive Dysfunction. Lancet (London, England). 1998;351(9106):857-61.

4.	Evered L, Silbert B, Knopman DS, Scott DA, DeKosky ST, Rasmussen LS, et al. Recommendations for the Nomenclature of Cognitive Change Associated with Anaesthesia and Surgery-2018. Anesthesiology. 2018;129(5):872-9. http://doi.org/10.1097/ALN.0000000000002334

Reviewer #2: Overall, this manuscript is well-written and includes a good foundation of information and methodology. To improve the paper further, I recommend adding more details, particularly in the methodology and discussion sections. For example, acupuncture, cognitive training, and transcutaneous electrical acupoint stimulation are mentioned in the intervention types but there are no additional details about them even in the discussion section. Furthermore, the manuscript talks about routine care and sham groups but provides no additional details. It would be helpful to discuss the different types of biases associated with meta-analysis and provide reasoning and remedies. Additionally, something like this "The study will use a node-splitting model to evaluate local inconsistencies at the network level." will not resonate with a lot of readers; a little more explanation will help them conceptualize the method and better understand the analysis. Expanding on areas like these will enhance the clarity and depth of your work, making it more accessible to a wider audience and offering greater insight into the findings.

**Response:** We extend our heartfelt gratitude for the affirmation and constructive feedback provided. In deference to your suggestions, we have implemented the subsequent revisions to the manuscript.

(1) We further elaborate on intervention methods (MA, EA, TEAS, and CT) and control methods (usual care and sham) in the Methods and Discussion section. Page 9, lines 150-157; Page 19, lines 291-306；

(2) The protocol will explore the sources of bias risk through subgroup analysis, sensitivity analysis and other methods. Page 20, lines 311-316；

(3) The protocol has been elaborated on the methodological details of "Assessment of Inconsistency," which also provides more specific guidance for our subsequent work. Page 17, lines 253-259.

Reviewer #3: I have observed significant improvements in the manuscript's structure and the conceptualization of terms related to cognitive dysfunction. I am confident that this paper merits consideration for publication in one of the editions of the esteemed PlosOne journal.

**Response:** Thank you very much for your affirmation and feedback.
